# Spinal cord injury: a study protocol for a systematic review and meta-analysis of microRNA alterations

**DOI:** 10.1186/s13643-022-01921-8

**Published:** 2022-04-05

**Authors:** Seth Tigchelaar, Zihuai He, Suzanne Tharin

**Affiliations:** 1 Department of Neurosurgery, Stanford University Medical Center, Stanford, CA USA; 2 Department of Neurology, Stanford University Medical Center, Stanford, CA USA; 3 Division of Neurosurgery, VA, Palo Alto, CA USA

## Abstract

**Background:**

Spinal cord injury (SCI) is a devastating condition with no current neurorestorative treatments. Clinical trials have been hampered by a lack of meaningful diagnostic and prognostic markers of injury severity and neurologic recovery. Objective biomarkers and novel therapies for SCI represent urgent unmet clinical needs. Biomarkers of SCI that objectively stratify the severity of cord damage could expand the depth and scope of clinical trials and represent targets for the development of novel therapies for acute SCI. MicroRNAs (miRNAs) represent promising candidates both as informative molecules of injury severity and recovery, and as therapeutic targets. miRNAs are small, regulatory RNA molecules that are tissue-specific and evolutionarily conserved across species. miRNAs have been shown to represent powerful predictors of pathology, particularly with respect to neurologic disorders.

**Methods:**

Studies investigating miRNA alterations in all species of animal models and human studies of acute, traumatic SCI will be identified from PubMed, Embase, and Scopus. We aim to identify whether SCI is associated with a specific pattern of miRNA expression that is conserved across species, and whether SCI is associated with a tissue- or cell type-specific pattern of miRNA expression. The inclusion criteria for this study will include (1) studies published anytime, (2) including all species, and sexes with acute, traumatic SCI, (3) relating to the alteration of miRNA after SCI, using molecular-based detection platforms including qRT-PCR, microarray, and RNA-sequencing, (4) including statistically significant miRNA alterations in tissues, such as spinal cord, serum/plasma, and/or CSF, and (5) studies with a SHAM surgery group. Articles included in the review will have their titles, abstracts, and full texts reviewed by two independent authors. Random effects meta-regression will be performed, which allows for within-study and between-study variability, on the miRNA expression after SCI or SHAM surgery. We will analyze both the cumulative pooled dataset, as well as datasets stratified by species, tissue type, and timepoint to identify miRNA alterations that are specifically related to the injured spinal cord. We aim to identify SCI-related miRNA that are specifically altered both within a species, and those that are evolutionarily conserved across species, including humans. The analyses will provide a description of the evolutionarily conserved miRNA signature of the pathophysiological response to SCI.

**Discussion:**

Here, we present a protocol to perform a systematic review and meta-analysis to investigate the conserved inter- and intra-species miRNA changes that occur due to acute, traumatic SCI. This review seeks to serve as a valuable resource for the SCI community by establishing a rigorous and unbiased description of miRNA changes after SCI for the next generation of SCI biomarkers and therapeutic interventions.

**Trial registration:**

The protocol for the systematic review and meta-analysis has been registered through PROSPERO: CRD42021222552.

**Supplementary Information:**

The online version contains supplementary material available at 10.1186/s13643-022-01921-8.

## Background

In the last four decades, improvements in medical, surgical, and rehabilitative care have increased the quality of life and extended the life expectancy of individuals with spinal cord injury (SCI)—once considered an imminently fatal condition [1]. However, while management has improved, there are no therapies for SCI that have demonstrated convincing neurologic benefit in large-scale clinical trials [2–6]. There are, therefore, urgent unmet needs for the preclinical scientific development of novel therapeutic strategies and for the subsequent clinical validation of these treatments in human trials.

MicroRNAs (miRNAs) hold great promise to underlie novel treatment strategies for SCI. miRNAs are small, noncoding RNAs, approximately 22 nucleotides in length, that regulate at least 30% of all protein coding genes [7]. Many miRNAs have been identified in the central nervous system (CNS) [8–11], with several showing CNS-specific expression [12]. Using microarray analysis, real-time PCR, and in situ hybridization, Bak et al. found 44 miRNAs that were more than threefold enriched in the brain or spinal cord [8] and Liu et al. found that nearly 80% of detected miRNAs were expressed in the adult rat spinal cord [13]. miRNAs also show cell type-specific expression in the brain and spinal cord, with specific miRNAs being expressed in neurons [12, 14], astrocytes [15, 16], and oligodendrocytes [17]. We have shown that miRNAs are required for the development and possibly the evolution of the corticospinal system [18], injury to which results in paralysis in SCI [19].

We and others have also shown that miRNA levels in cerebrospinal fluid (CSF) and blood serum are specifically altered in SCI in a severity-dependent fashion, both in humans [20], and in animal models [13, 21]. miRNAs are furthermore differentially altered in chronic SCI patients undergoing active exercise regimes, compared to sedentary patients [22]. miRNAs are promising biomarkers of injury severity, and by implication—recovery and response to treatment [23–25] due to their regional abundance, specific developmental requirements, and altered levels following SCI. miRNAs represent promising therapeutic targets for CNS injuries and disease, given their intimate involvement in the development and injury of the relevant circuitry, their ability to cross barriers and membranes, and their potential for rapid transition from bench to bedside [26, 27].

To inform the next stage of miRNA biomarker and preclinical therapeutic interventions, a comprehensive and systematic understanding of the disparate existing data would allow development of unified models and testable hypotheses. There has not been a meta-analysis of miRNA changes in the setting of SCI that includes the results of recent comprehensive human trials. Here, we present a protocol outlining our approach to a meta-analysis of the current literature, in which we will provide a comprehensive review, interpretation of results, and discussion of the methodology used to document, quantify, and analyze miRNA alterations in the setting of SCI.

## Methods/design

### Protocol registration and standard reporting

**Fig. 1:**
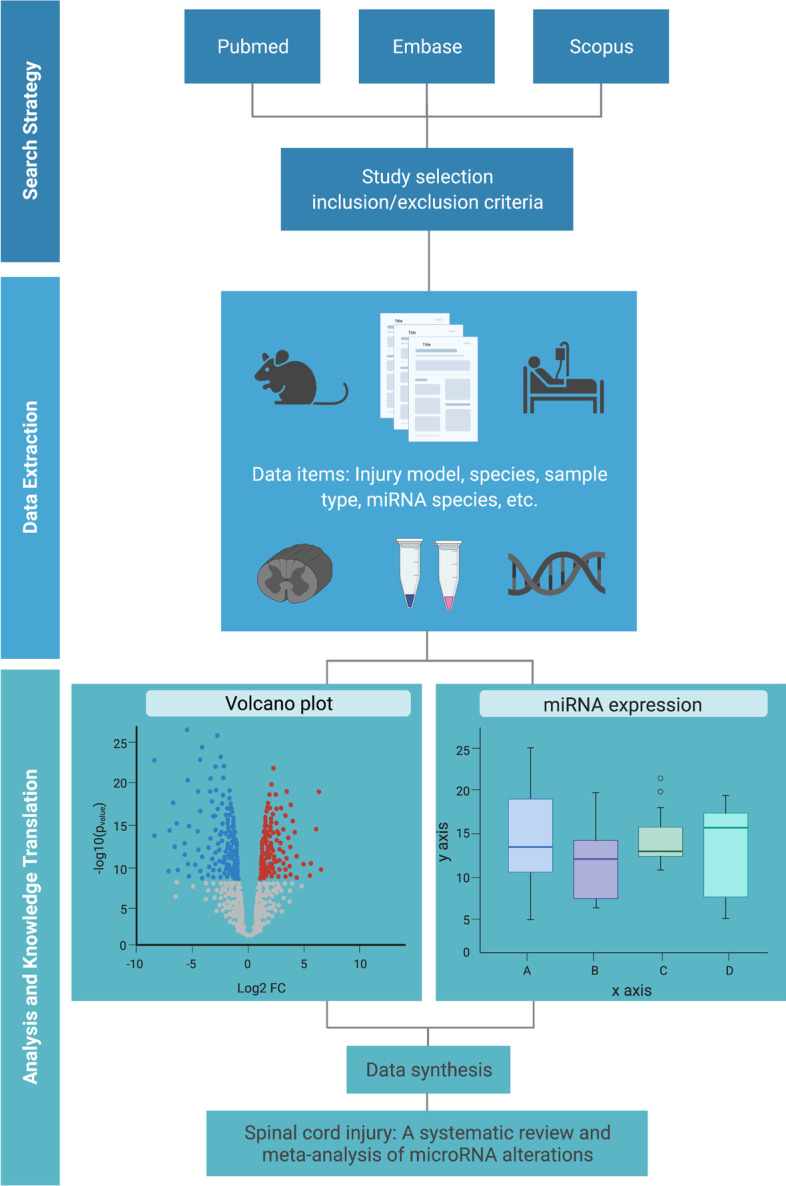
Schematic overview of approach to systematic review and meta-analysis

For the preparation and development of this protocol, we followed the checklist provided by the Preferred Reporting Items for Systematic Reviews and Meta-Analyses Protocols (PRISMA) [28] (see Additional file 1). The protocol for the systematic review and meta-analysis has been registered through PROSPERO: CRD42021222552. The schematic overview of approach to the systematic review and meta-analysis is represented in Fig. 1.

### Research question

We will conduct a systematic review and meta-analysis of studies evaluating miRNA alterations following SCI. The specific research questions we aim to answer with the meta-analysis presented here are:

1. Is SCI associated with a specific pattern of miRNA expression that is conserved across species?
2. Is SCI associated with a tissue- or cell type-specific pattern of miRNA expression?
3. Which SCI-associated miRNA alterations are consistent across, and which are unique to, specific injury models?

### Information sources and search strategy

Studies investigating miRNA alterations in all species of animal models of SCI and human studies will be identified from PubMed, Embase, and Scopus (including and up to 12/04/2020) (Table 1). Our search strategy was developed in consultation with an expert librarian and information specialist in systematic reviews.

**Table 1:** .Search strategy. The electronic databases PubMed, Embase, and Scopus will be search using the query shown in each column

PubMed Query	Embase Query	Scopus Query
"MicroRNAs"[Mesh] or "RNA, Untranslated"[Mesh] OR "untranslated rna"/exp OR "microrna*"[tw] OR "mirna*"[tw] OR "micro rna*"[tw] OR "mir"[tw] OR "non coding rna*"[tw] OR ncrna*[tw] OR "non protein coding rna*"[tw] OR "noncoding rna*"[tw] OR "untranslated rna*"[tw] or "npcrna*"[tw] or "non translated rna*"[tw] or "non peptide coding rna*"[tw]	'microrna'/exp OR 'untranslated rna'/exp OR 'microrna*':ti,ab,kw OR 'mirna*':ti,ab,kw OR 'micro rna*':ti,ab,kw OR 'mir':ti,ab,kw OR 'non-coding rna*':ti,ab,kw OR ncrna*:ti,ab,kw OR 'non protein coding rna*':ti,ab,kw OR 'noncoding rna*':ti,ab,kw OR 'untranslated rna*':ti,ab,kw OR 'npcrna*':ti,ab,kw OR 'non translated rna*':ti,ab,kw OR 'non peptide coding rna*':ti,ab,kw	( TITLE-ABS-KEY ( "spinal cord trauma*" OR "traumatic myelopath*" OR "spinal cord injur*" OR "spinal cord transection*" OR "spinal cord laceration*" OR "post-traumatic myelopath*" OR "spinal cord contusion*" ) ) AND ( TITLE-ABS-KEY ( "microrna*" OR "mirna*" OR "micro rna*" OR "mir" OR "non-coding rna*" OR ncrna* OR "non protein coding rna*" OR "noncoding rna*" OR "untranslated rna*" OR "npcrna*" OR "non translated rna*" OR "non peptide coding rna*" ) )
AND		
"Spinal Cord Injuries"[Mesh] or "Spinal Cord Trauma*"[tw] or "Traumatic Myelopath*"[tw] or "Spinal Cord Injur*"[tw] or "Spinal Cord Transection*"[tw] or "Spinal Cord Laceration*"[tw] or "Post-Traumatic Myelopath*"[tw] or "Spinal Cord Contusion*"[tw]	'spinal cord injuries'/exp OR ‘spinal cord trauma*’:ti,ab,kw or 'traumatic myelopath*':ti,ab,kw OR 'spinal cord injur*':ti,ab,kw OR 'spinal cord transection*':ti,ab,kw OR 'spinal cord laceration*':ti,ab,kw OR 'post-traumatic myelopath*':ti,ab,kw OR 'spinal cord contusion*':ti,ab,kw	

Our PubMed search strategy is as follows: “MicroRNAs”[Mesh] or “RNA, Untranslated”[Mesh] OR “untranslated rna”/exp OR “microrna*”[tw] OR “mirna*”[tw] OR “micro rna*”[tw] OR “mir”[tw] OR “non coding rna*”[tw] OR ncrna*[tw] OR “non protein coding rna*”[tw] OR “noncoding rna*”[tw] OR “untranslated rna*”[tw] or “npcrna*”[tw] or “non translated rna*”[tw] or “non peptide coding rna*”[tw] AND “Spinal Cord Injuries”[Mesh] or “Spinal Cord Trauma*”[tw] or “Traumatic Myelopath*”[tw] or “Spinal Cord Injur*”[tw] or “Spinal Cord Transection*”[tw] or “Spinal Cord Laceration*”[tw] or “Post-Traumatic Myelopath*”[tw] or “Spinal Cord Contusion*”[tw].

Our Embase search strategy is as follows: “microrna”/exp OR “untranslated rna”/exp OR “microrna*”:ti,ab,kw OR “mirna*”:ti,ab,kw OR “micro rna*”:ti,ab,kw OR “mir”:ti,ab,kw OR “non-coding rna*”:ti,ab,kw OR ncrna*:ti,ab,kw OR “non protein coding rna*”:ti,ab,kw OR “noncoding rna*”:ti,ab,kw OR “untranslated rna*”:ti,ab,kw OR “npcrna*”:ti,ab,kw OR “non translated rna*”:ti,ab,kw OR “non peptide coding rna*”:ti,ab,kw AND “spinal cord injuries”/exp OR “spinal cord trauma*”:ti,ab,kw or “traumatic myelopath*”:ti,ab,kw OR “spinal cord injur*”:ti,ab,kw OR “spinal cord transection*”:ti,ab,kw OR “spinal cord laceration*”:ti,ab,kw OR “post-traumatic myelopath*”:ti,ab,kw OR “spinal cord contusion*”:ti,ab,kw.

Our Scopus search strategy is as follows: ( TITLE-ABS-KEY (“spinal cord trauma*” OR “traumatic myelopath*” OR “spinal cord injur*” OR “spinal cord transection*” OR “spinal cord laceration*” OR “post-traumatic myelopath*” OR “spinal cord contusion*” ) ) AND ( TITLE-ABS-KEY ( “microrna*” OR “mirna*” OR “micro rna*” OR “mir” OR “non-coding rna*” OR ncrna* OR “non protein coding rna*” OR “noncoding rna*” OR “untranslated rna*” OR ”npcrna*” OR “non translated rna*” OR “non peptide coding rna*” ) ).

### Eligibility criteria

The inclusion criteria for this study will be as follows: (1) studies published anytime, (2) including all species, and sexes with acute, traumatic SCI, (3) relating to the alteration of miRNA after SCI, using molecular-based detection platforms including qRT-PCR, microarray, and RNA-sequencing, (4) Including statistically significant miRNA alterations in tissues, such as spinal cord, serum/plasma, and/or CSF, and (5) studies with a SHAM surgery group. The exclusion criteria for this study will be as follows: (1) studies not relating to acute, traumatic SCI, 2. Non-English articles, (3) in vitro, ex vivo, in silico studies, (4) non-peer-reviewed articles and conference abstracts, (5) studies focused solely on differential expression of miRNA in chronic SCI, defined here as > 7 days post-SCI, and (6) case reports or case series.

### Study selection

All original studies will be eligible for inclusion. Two reviewers will screen the imported studies using Covidence [29], reading title and abstract, and exclude studies that do not meet the inclusion criteria. At the selection phase, two reviewers will independently include studies that meet all eligibility criteria. Any discrepancies will be resolved by consensus. The results of study selection will be presented using a PRISMA flow diagram.

### Inclusion and exclusion criteria

#### Animals/population

All species and sexes of animal acute, traumatic SCI models and studies including human patients with acute, traumatic will be included in this analysis. Any in vitro studies, ex vivo studies, in silico studies, and polytrauma studies that do not include *in vivo* analysis will not be included.

#### Intervention/exposures

All studies including animal models of acute, traumatic SCI (including, but not limited to, contusion, transection, traumatic) and human studies of acute, traumatic SCI will be included. Any study of CNS injury not including the spinal cord will not be included.

#### Comparator group

All studies with the inclusion of a SHAM surgery comparator or control group (group without SCI) will be included. Any study without a SHAM-operated control group will not be included.

#### Outcome measures

All studies including the detection of miRNAs and their alteration, in spinal cord parenchyma, blood, plasma/serum, or CSF, in response to acute, traumatic SCI or SHAM surgery, within at least 7 days will be included. Any study that does not report miRNA changes within 7 days of SCI will not be included. Any study that does not report the direction of change of miRNA in response to SCI or SHAM surgery will not be included. Data regarding miRNA presence and alteration at each time point will be recorded as up- or downregulated, reported fold-changes, and associated statistical significance. miRNA alterations between post-SCI groups and post-SHAM surgery groups at respective time points will be compared in order to identify changes in miRNA specifically relating to the injured spinal cord.

### Data extraction and data items

Data will be extracted by two reviewers. Data parameters collected will include but will not be limited to (1) author/year, (2) study title, (3) study model, (4) study subjects (species, sex, sample size), (5) SCI model (contusion, transection), (6) miRNA detection platform (RT-PCR, microarray, sequencing), (7) miRNA direction and/or magnitude of change, (8) normalization method, (9) miRNA target (predicted and/or validated), (10) reported *p* value and/or nominal p-value (the nominal *p* value is a calculated observed significance based on a given statistical model) of each miRNA change, and 11. Risk of bias. When values are not explicitly provided, the extraction of statistical data from graphs will be performed using the graphical data extraction application, WebPlotDigitizer (Version 4.4) [30].

#### Bibliographic information

Information including PubMed ID, Embase ID, authors, and year of publication will be collected.

#### Subject characteristics

Information including species, sex, and age will be collected. If numerical values are presented as an interval, then the mean value will be calculated and reported instead.

#### Injury parameters

Information including the injury model (contusion, transection, etc.), injury severity, injury location (cervical, lumbar, thoracic), and timepoint of sample collections will be collected. Injury severity will be categorized as “mild” or “severe” SCI. For studies using a contusion model, force of injury (measured in kDynes) will be divided into 2 groups with the lower 50^th^ percentile classified as “mild”, and the upper 50th percentile classified as “severe.” Studies using a transection model will be classified as “severe” given that the spinal cord is completely transected. For clinical studies involving patients with SCI, the International Standard for Neurological Classification of Spinal Cord Injury (ISNCSCI) exam will be used to classify injury severity.

#### Differential miRNA expression

Information including the miRNA extraction method, library generation, and detection platform will be collected. Changes in miRNA expression will be recorded as up- or downregulated following SCI. Where fold-change, relative change, or other quantitative descriptions of changes are described, these data will be included.

### Data analysis and synthesis

We will present results using narrative and graphical methods, where study results are obviously heterogeneous by visual inspection, where results from different studies are presented using statistical measures that we cannot combine, for multivariable prediction models with few studies, or if there is insufficient representation of individual species, or species-specific miRNAs.

Where there are sufficient studies allowing extraction of results in the same format with reasonable homogeneity to allow summary by meta-analysis, we will apply a random effects meta-regression, which allows for within-study and between-study variability. This analysis will first be performed on the pooled data set to identify miRNA changes relating to SCI, while remaining agnostic to species, tissue type, or time point. In addition to the pooled dataset, analyses will seek to identify SCI-related changes in miRNA after stratifying by species, tissue type, and timepoint. The covariates will include, but will not be limited to: species, sex, injury model, injury level (cervical/thoracic/lumbar), injury severity, tissue type, timepoint, miRNA detection platform, and normalization strategy. To integrate studies where only the fold-change and *p* value are available, we will additionally apply a *p* value based meta-analysis using an inverse normal method, allowing us to use the data from as many studies as possible. The method will incorporate the direction of effect, the magnitude of signal, and the sample size. Unlike a meta-regression which aggregates exact effect sizes, the *p* value based method is an aggregation of association signals and the direction of effects (up/downregulation of miRNA). It only requires scale-free statistics as input, including *p*-value *p*_*i*_, sample size *n*_*i*_ and direction of effect *δ*_*i*_ per study. Therefore, it can integrate more studies where the effect size was not reported. The output is an aggregated Z-score/*p*-value, instead of a specific meta-analysis effect size which would be challenging to interpret across populations in which the effect sizes can be different in scale. Specifically, the meta-analysis Z-score can be computed as

$$Z=\frac{1}{\sqrt{\sum_i{w}_i^2}}\sum_i{w}_i{Z}_i,$$

where *Z*_*i*_ = Φ^−1^(*p*_*i*_/2) ∗  *sign* (*δ*_*i*_); ${w}_i=\sqrt{n_i}$. A meta-analysis *p*-value can be computed subsequently. Nominal *p*-values for all miRNAs will be adjusted using the Benjamini-Hochberg FDR-controlling method for multiple hypothesis testing.

We will use *I*^2^ to quantify between study heterogeneity. The *I*^2^ Index will be interpreted according to the Cochrane handbook [31]. When the *I*^2^ index is less than 40%, the statistical heterogeneity is not significant. The advantages and disadvantages of the respective study designs (injury models, miRNA detection platforms, normalization strategies, etc.) will also be discussed. Whenever a control group serves more than one experimental group, we will correct the total number of control animals in the meta-analysis by dividing the number of animals in the control group by the number of treatment groups served. Where applicable, Holm-Bonferroni correction for testing multiple subgroup analyses will be performed.

### Interpretation of results

In the interpretation of our results, the *p* value based meta-analysis proposed here represents an aggregation of association signals and their direction of effect (i.e., upregulation/downregulation of miRNA). The output, as an aggregated adjusted *p* value, will be interpreted as an indication of statistical significance that the specific miRNA in question was significantly associated with the conditions tested—that is, where we stratify by timepoint and tissue type, our interpretation would be: “spinal cord injury is significantly associated with increased/decreased expression of the specific miRNA within the spinal cord parenchyma at 24 h post-injury”. Studies investigating RNA-seq expression data across species have shown that the inter-study distances between homologous tissues of different species were smaller than intra-study distances among different tissues (within the same species), enabling informative meta-analyses [32–34]. Results will be displayed as summary statistics, describing the phenotypes of studies themselves, including number of studies using specific species, injury types, RNA detection modalities, timepoints, and tissue types. Individual miRNA results will be reported as tables with number of studies reporting each miRNA along with its aggregated adjusted p-value. All results, including those that are not statistically significant will be provided as a resource in supplemental data.

### Risk of bias

The use of SYRCLE’s risk of bias tool and the CAMARADES checklist for study quality will be included to assess risk of bias and study quality by two reviewers [35, 36]. Any disagreement will be resolved by consensus. Additionally, publication bias will be visually inspected with funnel plots.

## Discussion

miRNAs represent promising molecules to address two primary goals: developing diagnostic and prognostic tools for SCI and creating novel therapeutics. The mechanisms involved in the pathogenesis of SCI include a primary mechanical injury (impact) and a secondary injury induced by multiple subsequent biological processes, including a local inflammatory response, cytotoxicity, apoptosis, and demyelination [37, 38]. In addition to local inflammation, a systemic inflammatory response, inducing organ damage, has been shown to occur following SCI [39]. Although altered gene expression significantly contributes to the pathogenesis of secondary SCI [38], the regulatory networks that control it are not well understood. One aspect of the complex nature of secondary SCI could relate to gene regulation by miRNAs [16, 40–42]. As potential biomarkers of a pathological state, miRNAs are not constrained by cell membranes and communicate in extracellular fluids as free-floating miRNA [43] or within exosomes [44], and are considered stable, with relatively long half-lives of greater than 24 h [45]—as such, miRNA are appealing candidates for monitoring CNS pathophysiology related to SCI.

This is a critical juncture in the nascent field of miRNA biology in SCI, at which a synthesis and rigorous objective analysis of the collective data amassed over the last two decades with respect to miRNA changes after SCI will significantly inform future investigations. A systematic review and meta-analysis will be relevant to the SCI community and provide a comprehensive resource of robust miRNA changes across a spectrum of SCI paradigms with various injury models, species, tissues, and time points. This systematic review and meta-analysis will seek to serve as a resource by establishing a thorough, rigorous, and unbiased understanding of spinal cord injury-specific miRNA and the target pathways regulated by these miRNAs for the next generation of biomarkers and therapeutic interventions.

### Strengths and limitations

Here we propose a systematic approach to search for articles in three major databases (PubMed, Embase, and Scopus), in which screening for eligible articles will be performed by two independent reviewers. This review will provide a comprehensive analysis of miRNA changes following SCI and assist researchers and clinicians alike in the pursuit of biomarkers and novel therapeutics for SCI. The main limitations of this study will be the heterogeneous nature of the studies included. A major goal of this meta-analysis is to identify cross-species, clinically relevant changes in miRNA due to SCI, therefore it will be critical to combine experimental animal models and human patients with SCI. However, we also propose a stratified analysis by species, tissue type, and time point in order to address this heterogeneity. We acknowledge that there will be additional heterogeneity in miRNA detection platforms, and have limited our search to include only molecular detection platforms including RT-PCR, microarray, and RNA-Seq. An additional limitation is the exclusion of articles not indexed in PubMed, Embase, or Scopus, and of those articles that might not include relevant keywords or phrases used in this search.

## Supplementary Information

**Additional file 1.** PRISMA-P 2015 Checklist.

## Data Availability

All data generated and/or analyzed in this study will be included in the final published article and its supplementary files.

## Abbreviations

CNS: Central nervous system
CSF: Cerebrospinal fluid
miRNA: MicroRNA
SCI: Spinal cord injury
